# Richmond Academy of Medicine and Surgery

**Published:** 1896-10

**Authors:** 


### SOCIETY REPORTS.

### RICHMOND ACADEMY OF MEDICINE AND SURGERY.

*Regular Meeting, August* 11, 1896, *Hall of Y. M. C. A.*

Dr. Landon B. Edwards, President, in the chair. Dr. Mark W. Peyser, Secretary and Reporter.

### DISLOCATION OF THE ARCH OF THE FOOT.

Dr. J. W. Henson reported a case of dislocation of the arch of the foot. Woman, on June 9th, while crawling along a shed to reach a window, fell a distance of fifteen feet, alighting on her feet, dislocating the arch of the right foot. The foot turned outward like that of a negro, and the internal cuneiform could be felt in the middle of it. She had made a wooden sandal to support the arch. There was no injury elsewhere, except rupture of the internal lateral ligament of the left knee.

Dr. M. D. Hoge, Jr., reported a similar case. Man, aged 20, while in a somnambulastic state, climbed out of a fourth story window, falling on his feet. The arch of one foot was completely broken, and it spread out flat—a protuberance being discernible in the middle. He had made a leather wedge, which was put in the sole of the shoe, the thick edge inward. The man now walks as well as any one.

### THE ANTI-TUBERCLE SERUM TREATMENT OF CONSUMPTION.

Dr. J. Allison Hodges mentioned his experience with this treatment in several cases, not all of them having been benefited by it, but referred especially to the following case: He was called on May 10th to see Mr. A., aged 42, who was confined to bed—temperature, 102.5; respiration, 26; great difficulty in breathing, attended with an incessant cough and expectoration, and intermittent pain in right side. On examination, he found consolidation of the lower two-thirds of the right lung, with a suspected abscess forming. The sputum was subjected to microscopic examination, and revealed the presence of tubercle bacilli in large numbers. In response to questions as to the former history of this case, it was learned that his family history was good; that on January 17th, of this year, he had suffered from a slight attack of facial paralysis, and again on April 2d he had experienced a slight attack of hemiplegia on the right side. From both of these attacks he had rallied, and now nothing was noticeable, except a slight deviation of the tongue and weakness in the right leg. Further examination revealed an obscure history of syphilis. The past improvement in his nervous system was due to the use of the iodides in moderate dosage. In view of these facts, a diagnosis of "mixed infection" was made, and he was again put upon specific treatment in largely increased doses.

On the fifth day the abscess ruptured, accompanied with intense fetor, but there was no abatement of the fever or cessation of the cough. In fact, the case seemed rapidly progressing towards a fatal termination. For five days longer, the specific medication, together with symptomatic treatment, was continued, but without perceptibly favorable results.

It was then determined to institute the anti-tubercle treatment, and it was commenced on May 25th, with 15 minims of the Paquin "anti-tubercle serum," and continued daily, increasing 5 minims each day until 25 minims were given. At the time of commencing this treatment, the patient was almost exhausted, and in the opinion of several physicians who saw him, his case was considered all but hopeless. After the third day of treatment, there was noticeable a slight reduction in the temperature, and on the fifth day the patient was enabled to secure a comfortable night's rest, the cough having diminished to a great extent, and the exhausting night sweats having almost entirely ceased.

After the eighth day of treatment the improvement was still greater, the temperature becoming normal, the appetite returning, and the cough and night sweats almost entirely abating.

The treatment was now continued at 20 minims almost daily, for on two occasions when the dose was gradually increased to 40 minims, an irritative febrile condition was induced, and at the same time an increase of albumen in the urine and indications of an erythematous eruption were noticed. In two weeks the patient was enabled to sit up for several hours at a time, and was altogether comfortable.

The treatment was continued till July 1st., having consumed a little more than one month, and on the following day the patient walked a mile and stood for four hours in the streets, reviewing a parade. He is still under observation, and is now taking ninety drops of a saturated solution of potassium iodide daily.

The happy termination of this case, it is admitted, is an exceptional one, but it is illustrative beyond doubt of the efficacy of the serum treatment, even in some cases of mixed infection, which are not the accepted cases for its exhibition.

Some of the features noted in this case worthy of observation are:

1. The speedy reduction in temperature—having improved after the fifth injection, and becoming normal after the eighth.

2. The improvement in the cough and expectoration, the fetor ceasing entirely after the tenth injection.

3. The abatement of the night sweats, and their subsequent cessation at the expiration of the second week.

4. The return of the appetite, and a gain up to the present time of eighteen pounds in weight.

5. The improvement in the consolidated lung tissue, though this condition cannot be said yet to be "cured," but the microscope reveals a continually decreasing number of bacilli present.

Dr. Virginius W. Harrison said that the case reported by Dr. Hodges recalled to his mind a case similar in some respects. A white man, aged 32, tubercular history, two deaths in his family, he himself having suffered from what was called scrofula in his early childhood—the scars of the removal of the glands now disfiguring his neck. The patient was then emaciated, appetite gone, night sweats, and a condition which looked like an early dissolution of the body. The patione said that he had suffered from shortness of breath and cough for several months—the shortness of breath increasing. On examination of his lungs, the right side showed that the apex was doing practically no work; the lower portion was consolidated. Temperature 102. Dr. Harrison advised him to go home and go to bed, but he declined to go, and went to work, and continued at work for two days, when he had a chill. On the third day he had another chill, and went to bed. The doctor saw him on the fourth day, when he said that, after the first chill, he suffered intense pain in the whole right side. Some hours after the second chill his cough increased a great deal, and soon a bloody, offensive pus was expectorated; during that twenty-four hours he spit out one quart of this material. This continued, so that in the next twenty-four hours he expectorated nearly a pint, and gradually diminished until six weeks had passed and it ceased. Dr. Harrison got a bottle of this material (which he showed to several members of the Academy), and got Mr. Hugh Blair to examine it for him. The examination revealed the nature of the material to be pus, mucus, and blood. Dr. Harrison did not have it examined for the tubercle bacillus, but with such a personal history and family history, he feels justified in concluding that the condition was due to tuberculosis.

Dr. Harrison remarked that he is aware that tuberculosis of a gland may be perfectly eliminated by removal or suppuration, when the tuberculosis is in the gland alone; and he is further aware that the same bacillus may be latent in other portions of the body, awaiting renewed excitation to renew its energy in its deadly work. This man was treated by inhalations of beechwood creosote, and given liquid peptonoids and tonics. He has recovered sufficiently from this spell to return to his former occupation. The future of this man, in his opinion, is most doubtful, but the advocates of the serum therapy seem unwilling to show their confidence in its use by dropping the drugs they have found in years past to be useful in the same way that serum therapy claims to aid in the diseases.

Dr. Harrison added that he would be glad to hear from Dr. Landon B. Edwards on this subject, as we know he has had a good many patients under treatment by this new anti-tubercle serum method.

Dr. James N. Ellis remarked that, in using anti-tubercle serum, it is well to look out for the development of an eruption. He ignored the first red blotches, and the man did not sleep for five days. Injection of three-fourths of a grain of morphine had no effect whatever in allaying the intense irritation.

The President said that, up to the present time, he had treated fourteen cases with Paquin's anti-tubercle serum. Two of these, referred to him by other physicians, are hopeless. In one of these two, whenever coughing results in free expectoration, the temperature is reduced to 99° or 100°, but in twenty-four hours it is up again. Cavernous respiration is so marked that, as was said, no hope could be held for recovery. The second of the two is even more hopeless. It is a constant matter of wonder how he lives. In three other cases there has been no material improvement, but neither have they grown worse. In the remainder there has been such improvement that two have been discharged, apparently cured, but with advice to return occasionally for inspection, as it were. One of these is a vocalist, who says she sings with the same power as before she was affected. The second is a medical student, who, since his discharge, has gone through a mild attack of pneumonia, which left some slight consolidation. The hot weather prevents, in a measure, carrying out all details of treatment.

Right here may be remarked that, with this record, it must be acknowledged this treatment is of great benefit.

Dr. Edwards said he never could have expected such good results from creosote and tonic treatment generally, as he has had in the nine cases, which include the two just cited. One of those discharged is using creosote internally. In one case, now much improved, death was expected in three months at most; and while he is not completely cured yet, the advance toward it is remarkable. In another case, the attack was in its incipiency, and creosote alone might have cured without the serum. Whenever the temperature is high, and remains so, no treatment is of service. Edson's treatment has not seemed to make any favorable impression.

The cases mentioned, in which fatal issue seems inevitable, are of mixed infection.

If Paquin's treatment had no curative value, its palliative qualities would alone render it remarkable—stopping night sweats, non-interference with appetite, if anything, increasing it, etc. In hot weather, cod-liver oil does more harm than good. If employing it, do so in the form of emulsion. When the stomach is enervated, it does as much damage as anything can.

Dr. Harrison: Have you used the serum treatment alone? If not, is it because you have not the necessary confidence?

Dr. Edwards replied that he employs accessory treatment, simply as in fighting he would employ both fists, not depending on the right solely. With so dangerous an enemy as tuberculosis, he is unwilling to do less than everything that promises good.

Dr. Hodges stated that he had used the serum treatment alone with benefit.

### ABSCESS OF PALM OF HAND FOLLOWING TYPHOID FEVER.

The Secretary reported the case of a boy convalescing from typhoid fever, who, two or three days after temperature reached normal, complained of pain in the right hand. Next day the pain was more severe, and swelling had occurred. Salicylates were prescribed, with the result, that, on the third day, pain had subsided, but swelling was found greatly increased. That afternoon information was received that the "hand had burst and corruption was pouring out." On investigation, this was found to be true, and further, that the pus had dissected its way clear to the tips of the fingers and down to the wrist, the whole hand and all the fingers being involved, except the thumb and thenar eminence. Incisions were made between the index and middle, and ring and little fingers, to facilitate drainage. Under the daily employment of hydrogen dioxide and carbolized water, pus formation is rapidly ceasing. Of course thrombus was diagnosed, but the wonder is at the rapid formation of pus.

---

*Regular Meeting, August* 25, 1896, *Hall of Y. M. C. A.*

Dr. Landon B. Edwards, President, in the chair. Dr. Mark W. Peyser, Secretary and Reporter.

Dr. J. Allison Hodges read a paper on

THE NOT USUAL EFFECTS OF THE BROMIDES. (See page 529.)

Dr. J. N. Upshur remarked that Dr. Hodges' paper was one of great value to the Academy and the profession at large. He entirely agreed with the views expressed in it. For many years he has believed that the profession is as much too prone to give the bromides as morphine. He was peculiarly in sympathy with the statement as to the indiscriminate use by the profession and laity of the various proprietary preparations containing these agents. He has been in the habit of prescribing the bromides with more or less frequency for several years, because he did not wish to prescribe them empirically, and because of their effect on the circulation. He hoped the article would be widely read, and that the profession would heed the warning given.

Dr. Wm. S. Gordon agreed with both gentlemen preceding him. Of course, it is routine practice to give the bromides in epilepsy. He narrated the case of an epileptic girl who, after taking considerable doses, developed all the prominent symptoms mentioned by Dr. Hodges; and at the time, he wondered how far they were due to the medicine or the disease. He asked if, under these circumstances, had Dr. Hodges substituted hydrobromic acid, and, if so, the symptoms, when pushed to the farthest point? He has used hydrobromic acid with good effect, but he had never continued it long enough to contrast its effects with those of the bromide salts.

The President agreed with most of the points made by Dr. Hodges; and yet he could not allow the enthusiasm occasioned by the valuable paper to overshadow the good results often obtained from the use of the bromides. He did not know how we could manage without them. It was not usual, in his experience, for patients to desire a continuance of their use; he wished to do away with the idea that there is a bromide habit, due to a desire on the part of the patient to take this agent. Large doses have their value in certain cases; but, ordinarily, these are not called for. As a rule, the bromide treatment is the most valuable for epilepsy. In the experience of those treating *petit mal*, it is of little value; but in the *grand mal*, it is of utmost value.

Suicidal and homicidal tendencies are noted, not in the cases where there is deliberation and cunning, but in the careless, somnambulistic, etc. At any rate, he was not prepared to say these tendencies came from bromism. The epileptic is often dangerous to himself and others, even without treatment by bromides.

Dr. Hodges, in closing the discussion, agreed with Dr. Edwards in the efficacy of the bromides in some cases, but limited their greatest utility to cerebral disorders from overaction. He differed with him, however, as to the danger of patients forming a "bromo habit," and cited the large and increasing use by the laity of the numerous bromo preparations, as was evidenced by their enormous consumption within the past few years. He doubted the necessity of the bromides or their ultimate benefit to the patient in the treatment of epilepsy, and cited instances to substantiate this assertion, claiming that other drugs, or even the bromides differently administered, would accomplish the same results. He had used hydrobromic acid as a sedative and hypnotic, and had found it useful; but had never pushed it to its physiological effects. It was probable, however, that under such conditions, it would prove an irritant to the gastric mucous membrane.

---

Reports of cases :

### CONVULSIONS AND HEMI-HYPERESTHESIA PRECEDING TYPHOID FEVER.

Dr. Upshur reported the case of a woman, aged 20. Two months ago he was sent for at night and found she had had a severe convulsion before his arrival. When seen, her mind was not entirely clear—was very nervous, presenting symptoms of a markedly hysterical condition. There had been much malaise for a week previous. The prominent symptom was hyperesthesia of the whole right half of the body and head, especially marked in the arm and leg. Simple touch would throw the group of muscles in the neighborhood into violent spasms. Along with this, for a week, there had been a regular rise and fall of temperature—99° in the morning to 101° in the afternoon. At the end of the week, the hyperesthesia seemed to subside, but she was nervous and sleepless; the tongue was heavily coated, pulse frequent, abdomen distended and tender, with gurgling in the right iliac fossa. In fourteen or fifteen days, symptoms of marked typhoid made their appearance. From that point the case was typical, with a tendency to development of coma vigil. Coma did supervene, and the paient died on the twenty-seventh day. Obstinate constipation persisted throughout the course of the disease. Every action was procured by an enema. The onset of the trouble was the most curious he had ever seen.

Dr. Upshur also reported the next case, concerning which he would like to have an expression of opinion. Woman, aged 40; seen ten days ago in consultation. She had been in delicate health for about a year, and there was obscurity as to her trouble. During last spring she was satisfied as to the existence of pregnancy. Taking some medicine given by a negress, she aborted and recovered. Later, she was seized with violent pain in the right groin, extending around the crest of the ilium down the thigh—being particularly painful in the knee, and paroxysmal in character, especially pronounced in the morning and afternoon. There were rises and falls of temperature at various times of the day, from 99° to 104-5° F.

During an attack, when first seen, he had found an overdistended bladder. The urine was albuminous, but no trouble ensued upon drawing it off.

Ten days ago she was removed to the Old Dominion Hospital, the temperature rising as a result two degrees, falling in a day or two.

The administration of chloroform did not effect a complete relaxation of the abdominal muscles, but a thorough examination of the pelvis revealed no trouble, unless, possibly, a pre-existent endometritis. A point just above Poupart's ligament on the right side showed evidence of tenderness. Per vaginam, he could approximate the fingers so that the thickness of the abdominal wall could be made out. Examination of the urine by Mr. Blair evidenced slight albumin, but was negative otherwise.

Query: What is the matter? The only pronounced symptom is the pain. She will not voluntarily change her position, and as a result, she has a bed-sore.

What is the cause of the rise of temperature? Under the use of turpentine the condition has improved somewhat. There may be an abscess of the kidney. He had suspected the pain to be hysterical. She had been taking a great deal of morphine—in one day a grain and a half hypodermically. Since being at the hospital the doctor has tried to do away with its use. The patient has taken several remedies for the relief of pain, usually falling back on morphine. As an experiment, a syringeful of pure water was injected, but in a half-hour complaint of great pain obliged administration of one-fourth grain of morphine. The doctor thought a great deal of the pain was due to the fear of it on being moved.

There were no sweats, no hectic; she is not sensitive to light pressure. Examination by her attending physician elicited great complaint of pain; but in ten minutes he could manipulate and not get much response.

Dr. Hodges wished to suggest that the first case was thrombus with suppuration, complicated or not by typhoid.

He thought the second case more than simple hysteria. If there were no disease of the pelvic organs, it might be neuralgia or neuritis with after-developed hysteria or fever.

Dr. Upshur replied that there was no neuritis.

### IS THERE A TYPHO-MALARIAL FEVER?

The Secretary reported a case, not because of uniqueness, but as contributing to the settlement of the much-mooted existence of typho-malarial fever.

T. H. P., male, aged 36, more than three weeks ago, was taken sick with what looked like cholera morbus. In four days a chill came on, followed by sweating and fever; thereafter, for four or five days, there were two and three chills at irregular intervals daily; the temperature one morning reaching 106°, falling to 102° upon the application of a cold bath. Thirty grains of hydrochlorate of quinine were administered in twelve hours, and as a result chills ceased, but there remained a persistent fever, which was stationary in the morning, rising in the afternoon, and accompanied by nearly all the symptoms which justified a diagnosis of typhoid fever. He entered his fourth week of sickness to-day, but his temperature has been normal for four or five days in the morning, with exacerbation in the afternoon. Chills came on again last Friday, and with the exception of Sunday, he has had them daily at irregular intervals, and sometimes two in twenty-four hours.

Dr. R. F. Williams made a blood examination and found absolutely no plasmodium, but a marked leucocytosis, which argues a favorable result.

Drs. Gordon and Deaton saw the case in consultation, and agreed that the trouble is pure typhoid.

Dr. Wm. S. Gordon said, regarding this case, that it was undoubtedly typhoid. If it had been typho-malaria, when the chills were stopped the fever should have ceased, and the blood examination further verified the diagnosis. He reported the case of a lady confined two weeks ago. He had ceased his attention, when on the twelfth day, she was seized by a violent chill at 4:30 p. m., and again at 11 p. m. Friday last, there was another at 11 a. m. Quartan fever being suspected, he gave quinine, but there ensued a chill at 11 p. m., followed by high fever and sweating. There was no trouble with the breast that morning; the lochia was free from odor, and everything relating to the uterus was normal. At night, the breast was affected and lead-water was advised. The next morning mammitis had come on—no suppuration. The breast trouble did not ensue until the fourth day. It was relieved. Yesterday, the patient was entirely free from fever. She was thoroughly cinchonized. Remembering the influence on the mind of the time of attacks, he endeavored to take hers from under its influence by mental diversion, anecdotes, etc.; but at 4:30 p. m. the chill came. If she had not been under the influence of quinine she would in all probability have had another last night. The case, a double quotidian of the quartan type, is one the doctor has never seen before.

As to the cause: The house is surrounded by flowers, grown over with vines; has, necessarily, decaying vegetation in the garden; flowers in the rooms. These, with moisture and heat, furnish all the etiology desired.

### FACIAL PARALYSIS FOLLOWING MUMPS, ENLARGED ONE-HALF OF THYROID, ETC.

Dr. Jacob Michaux described a case of facial paralysis. Girl, aged 17; personal history good. A short time ago he was told she had mumps. The left side of the face and lips were decidedly swollen, having the stiff appearance indicating loss of motion, which, indeed, was the case. There was tenderness under the ear, but he was struck by the absence of swelling in front of the ear (which usually occurs in mumps) and by the appearance of the lids, mouth and side of the face. The tissues on the affected side were tender, especially at the angle of the jaw.

On complaining of her throat, he examined it and found enlargement of the right lobe of the thyroid decided, but none of the left.

She was treated at home until acute symptoms subsided, with iodide of potassium and corrosive chloride of mercury. After she was able to be up and about, faradic electricity was applied to the affected side, and both it and galvanic, especially the latter, to the enlarged gland.

The doctor wished to know if there was any connection between the two conditions, or the parts. If so, what?

The girl was not positive as to the existence of thyroid enlargement before the facial symptoms. There was no sore throat, but some temperature.

### EXTRAUTERINE CAVITY.

Dr. Michaux's second case was an unusual condition of the uterine canal, which he has seen twice. Both were in cases of abortion—one at two, the other at four months. In the latter, on introduction of the finger into the os, he found a cavity, which, as well as he could make out, was three inches in diameter. At first, he thought he had a case of placenta previa, but on passing the finger through he discovered the fetus and placenta, which were duly delivered. In the other case the canal was smaller.

